# The Global Impact of Bacterial Antimicrobial Resistance: Addressing Gaps and Future Strategies

**DOI:** 10.1002/puh2.70229

**Published:** 2026-04-15

**Authors:** Safayet Jamil, Md. Golam Dostogir Harun, Mohammad Shahangir Biswas, Abdulrakib Abdulrahim, Neeru Chaudhary, Victor Abiola Adepoju, Hafiz T. A. Khan, Uthman Okikiola Adebayo

**Affiliations:** ^1^ Department of Public Health Daffodil International University Dhaka Bangladesh; ^2^ Department of Biochemistry and Biotechnology University of Science and Technology Chittagong Chittagong Bangladesh; ^3^ Department of Medical Microbiology, Faculty of Medicine and Health Sciences Universiti Putra Malaysia Serdang Selangor Malaysia; ^4^ Department of Public Health Delhi Pharmaceutical Sciences and Research University New Delhi India; ^5^ Department of HIV and Infectious Disease Jhpiego (An Affiliate of John Hopkins University) Abuja Nigeria; ^6^ College of Nursing, Midwifery and Healthcare University of West London Ealing London UK; ^7^ Oxford Institute of Population Aging University of Oxford Oxford UK; ^8^ Department of Medical Laboratory Services Federal University of Health Sciences Teaching Hospital Ila‐Orangun Nigeria; ^9^ Department of Digital Health Global Health Focus Africa Kigali Rwanda

**Keywords:** AMR surveillance systems, antimicrobial resistance (AMR), antimicrobial stewardship, Global Burden of Disease (GBD 2021), one health approach

## Abstract

Antimicrobial resistance (AMR) has become one of the most pressing threats to public health worldwide. According to the Global Burden of Disease 2021 report, AMR was linked to 4.71 million deaths, of which 1.14 million were directly caused by bacterial resistance. The impact is not felt equally, with low‐ and middle‐income countries (LMICs) carrying the greatest burden due to weak health systems, limited diagnostics and widespread misuse of antibiotics in both healthcare and agriculture. Self‐medication and poor regulatory oversight further accelerate the crisis. This article explores these challenges and stresses the urgent need for a coordinated ‘One Health’ approach that connects human, animal and environmental health. Strengthening surveillance, investing in new antibiotics and promoting responsible use through stewardship programs and community awareness are critical steps forward. Without decisive global action, AMR could claim over 8 million lives each year by 2050; yet, with timely interventions, especially in LMICs, up to 92 million deaths could be prevented, protecting future generations from its devastating consequences.

## Graphical abstract

Antimicrobial resistance remains a major global health threat, with the greatest burden concentrated in low‐ and middle‐income countries. Driven by antibiotic misuse, weak diagnostics, and human‐animal‐environmental transmission, the crisis demands a One Health response. Strengthened surveillance, stewardship, regulation, public awareness, and innovation are essential to reduce preventable deaths and slow the spread of resistance.

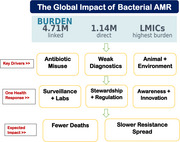

## 1 Introduction

Antimicrobial resistance (AMR) poses a severe threat to global public health. AMR poses substantial challenges to healthcare systems, economic stability and social welfare [1, 2]. If unaddressed, projections estimate that AMR could be associated with more than 8.2 million deaths annually by 2050, with a substantial proportion directly attributable to bacterial resistance, which would result in profound economic losses worldwide [3]. This commentary critically examines findings from the Global Burden of Disease (GBD) 2021 report, identifies current gaps in addressing AMR and proposes actionable strategies to mitigate its impact in line with global health initiatives like the World Health Organization's Global Action Plan on AMR.

### 1.1 Key Findings and Trends

This GBD report quantified AMR burden from 1990 to 2021 for 22 pathogens, 84 pathogen–drug combinations and 11 syndromes in 204 countries. It analysed all‐age and age‐specific deaths and disability‐adjusted life‐years attributable to and associated with AMR [3]. AMR‐related mortality varies notably by age and infection type. The report reveals significant regional disparities in AMR‐related mortality, as high burdens were observed in South Asia and sub‐Saharan Africa [3]. Although AMR mortality among children under 5 years has decreased by over 50% since 1990, the burden has shifted toward older adults, particularly those over 70, with an alarming 80% increase in deaths [3]. The increasing prevalence of multidrug‐resistant Gram‐negative organisms, particularly in low‐ and middle‐income countries (LMICs), signals an urgent need for targeted interventions [4].

### 1.2 Drivers of the AMR Crisis

AMR arises from a complex interplay of healthcare, agricultural, environmental and socio‐economic factors, with significant regional variation. Healthcare‐related drivers remain central to the AMR burden. Overprescription of antibiotics, frequent treatment of non‐bacterial infections and self‐medication are pervasive in LMICs, exacerbated by weak regulatory oversight and limited access to diagnostics [5, 6, 7]. For example, a study in Uganda reports that over 50% of the population engage in self‐medication, highlighting gaps in both community awareness and healthcare governance [6]. Although high‐income countries have established effective stewardship programmes, LMIC hospitals often face resource constraints that limit the implementation and monitoring of antimicrobial stewardship programs (ASPs) [8, 9].

Agricultural and environmental drivers further amplify AMR transmission. Extensive use of antibiotics for growth promotion and disease prevention in livestock and crop production contributes to the spread of resistant organisms from animals to humans through food chains, soil and water [10, 11]. Critically, most regional studies rely on cross‐sectional or modelling data with limited longitudinal validation, indicating a need for stronger empirical surveillance [12]. Environmental pathways, including contaminated wastewater, surface water and bioaerosols, facilitate horizontal gene transfer and cross‐species dissemination, yet remain underreported in LMIC research [13].

Surveillance and data limitations are another critical barrier to effective AMR control. Although global estimates, such as the GBD 2021 report, provide valuable insights, they rely heavily on modelling in regions with limited laboratory capacity, which may underestimate the true burden [3]. In many LMICs, surveillance systems are fragmented and lack standardization, restricting a timely detection of resistance trends and weakening evidence‐based policymaking [14].

Finally, governance and socio‐economic factors compound these challenges. Weak regulatory enforcement allows over‐the‐counter antibiotic sales, inconsistent agricultural controls and inequitable access to healthcare [7]. Urbanization, population growth and poverty intensify exposure risks and hinder adherence to recommended stewardship practices [15, 16].

### 1.3 Addressing Current Challenges and Opportunities for Interventions

Sustainable control of AMR requires a comprehensive One Health approach integrating human, animal and environmental health [17]. A key priority is strengthening surveillance systems through improved laboratory capacity, standardized antimicrobial susceptibility testing and coordinated data‐sharing platforms, particularly in resource‐limited settings [18]. Incentivizing the development of new antibiotics is also critical, especially for multidrug‐resistant Gram‐negative pathogens. Given the scientific and economic challenges facing the pharmaceutical industry, push–pull mechanisms, such as public–private partnerships and market entry rewards, are essential to stimulate innovation [19, 20].

Targeted interventions are needed across both community and healthcare settings [5, 6, 21]. At the community level, self‐medication and over‐the‐counter antibiotic sales remain major drivers of resistance, underscoring the need for stronger regulatory enforcement and public education [6]. Broad awareness campaigns through radio, television and social media can promote responsible antibiotic use [21], whereas behavioural strategies, such as pharmacy pledges, Short Message Service reminders and school‐based AMR education, can foster long‐term change [7, 21]. Governments and non‐governmental organizations should support outreach programs and farmer education to reduce non‐therapeutic antibiotic use in livestock under a One Health framework [10]. Improved hygiene, sanitation and safe disposal of unused antibiotics can further reduce community‐level risk.

Within healthcare settings, ASPs should be strengthened to optimize prescribing practices through monitoring, feedback and adherence to treatment guidelines [8, 9]. These efforts should be supported by improved infection prevention and control, access to rapid diagnostics and continued professional training to reduce inappropriate antibiotic use. Mandatory training and continuing medical education on AMR and stewardship are critical for healthcare professionals [22, 23]. The adoption of electronic prescription systems and expanded access to rapid diagnostics, including culture and sensitivity testing, can promote targeted therapy and reduce inappropriate antibiotic use [8]. Together, these coordinated actions provide a practical and scalable framework for mitigating AMR across sectors.

### 1.4 Future Directions and Recommendations

Expanding public awareness and strengthening national action plans should remain central to AMR control. Interventions must be context‐specific, particularly in LMICs, where regulatory enforcement, healthcare access and resource availability vary significantly [24, 25, 26, 27]. Integrated infection prevention and stewardship initiatives in Bangladesh and India demonstrate the importance of multisectoral approaches, whereas agricultural guidelines limiting non‐therapeutic antibiotic use in Kenya and Vietnam complement human health efforts under a One Health framework [28, 29].

Sweden's experience offers key lessons, including strict prescription regulations, robust stewardship and surveillance and active public engagement [30]. Although direct replication may be challenging, LMICs can adapt these principles through phased stewardship, sentinel surveillance and cost‐effective education, which integrates human, animal and environmental actions to achieve sustainable, scalable AMR control [31, 32].

Operationalizing a One Health approach requires coordinated implementation across sectors. Successful models from countries, such as Sweden, Thailand and South Africa, demonstrate the feasibility of combining surveillance, stewardship and public engagement into scalable AMR control strategies [33, 34]. Incentives for new antibiotic development combine push mechanisms (grants, partnerships like CARB‐X) and pull mechanisms (market entry rewards) [35]. Lastly, measurable outcomes, such as reporting coverage, data completeness and the number of antibiotics in development, provide a practical and adaptable roadmap for One Health AMR control [36, 37].

## 2 Conclusion

Addressing the AMR crisis necessitates immediate global collaboration. The GBD 2021 report reveals significant mortality rates, increasing regional inequalities and a growing burden on older populations in LMICs, jeopardizing healthcare and economic stability. A holistic One Health strategy must be implemented through enhanced surveillance, laboratory capabilities and data‐informed policies. Antimicrobial stewardship requires bolstering via electronic prescriptions, compulsory training, prescription audits and tighter regulations on over‐the‐counter antibiotics, along with improved infection control measures. Policies should also restrict non‐therapeutic antibiotic applications in agriculture, enhance farmer education and advocate for safer practices. Public awareness initiatives, educational programs in schools and digital outreach are crucial for diminishing self‐medication. Continued political dedication, investment in diagnostics and primary healthcare and renewed efforts in antibiotic innovation can profoundly influence the AMR trajectory and avert millions of preventable deaths in the future.

## Author Contributions

Safayet Jamil conceptualized this topic. Safayet Jamil, Md. Golam Dostogir Harun and Hafiz T. A. Khan supervised this project. Safayet Jamil, Md. Golam Dostogir Harun, Mohammad Shahangir Biswas and Victor Abiola Adepoju conducted literature review. Safayet Jamil, Abdulrakib Abdulrahim and Neeru Chaudhary wrote the first draft. Mohammad Shahangir Biswas, Abdulrakib Abdulrahim, Victor Abiola Adepoju, Hafiz T. A. Khan and Uthman Okikiola Adebayo revised and edited this manuscript. All authors approved the final version of the manuscript.

## Funding

The authors have nothing to report.

## Conflicts of Interest

The authors declare no conflicts of interest.

## Data Availability

Data sharing is not applicable to this article as no datasets were generated or analysed during the current study.
